# Spectrum of Mesenchymal–Epithelial Transition Aberrations and Potential Clinical Implications: Insights From Integrative Pancancer Analysis

**DOI:** 10.3389/fonc.2020.560615

**Published:** 2020-10-15

**Authors:** Juanni Li, Kuan Hu, Lei Zhou, Jinzhou Huang, Shuangshuang Zeng, Zhijie Xu, Yuanliang Yan

**Affiliations:** ^1^Department of Pathology, Xiangya Hospital, Central South University, Changsha, China; ^2^Department of Hepatobiliary Surgery, Xiangya Hospital, Central South University, Changsha, China; ^3^Department of Anesthesiology, Third Xiangya Hospital of Central South University, Changsha, China; ^4^Department of Oncology, Mayo Clinic, Rochester, MN, United States; ^5^Department of Pharmacy, Xiangya Hospital, Central South University, Changsha, China; ^6^National Clinical Research Center for Geriatric Disorders, Xiangya Hospital, Central South University, Changsha, China

**Keywords:** mesenchymal–epithelial transition factor, gene mutation, gene alteration, outcome, pancancer

## Abstract

**Background:**

The receptor tyrosine kinase mesenchymal–epithelial transition factor (MET) is frequently altered in cancers and is a common therapeutic target for cancers with MET variants. However, abnormal MET alterations and their associations with patient outcome across different cancer types have not been studied simultaneously. In this study, we try to fill the vacancy in a comprehensive manner and capture the full MET alteration spectrum.

**Methods:**

A total of 10,967 tumor samples comprising 32 cancer types from The Cancer Genome Atlas (TCGA) datasets were analyzed for MET abnormal expression, mutations, and copy number variants (CNVs).

**Results:**

MET abnormal expression, alteration frequency, mutation site distribution, and functional impact varied across different cancer types. Lung adenocarcinoma (LUAD) has most targetable mutations located in the juxtamembrane domain, and both high expression and amplification of MET are significantly associated with poor prognosis. Kidney renal papillary cell carcinoma (KIRP) harbored the third highest alteration frequency of MET, which was dominated by mutations. While most mutations were in the Pkinase_Tyr domain, a few were targetable. Pancreatic adenocarcinoma (PAAD) harbors very few alterations, but increased MET expression is associated with poor outcomes. Esophageal carcinoma (ESCA), stomach adenocarcinoma (STAD), and ovarian serous cystadenocarcinoma (OV) had similar characteristics: a high frequency of MET CNVs but relatively few MET mutations, and high MET expression associated with poor prognosis.

**Conclusion:**

This study provided significant and comprehensive information regarding MET abnormal expression, alterations (mutations and CNVs), and their clinical associations among 32 cancer types and offered insights into the full MET alteration spectrum and its implications for prognosis and treatment.

## Introduction

The human MET gene is located on chromosome 7q21–31 and encodes c-mesenchymal-epithelial transition factor (MET), which belongs to the family of receptor tyrosine kinases (1, 2). MET is a well-characterized oncogene and is a critical therapeutic target in several cancers (3). It is frequently activated in human tumors by various mechanisms, such as mutations, amplification, and overexpression (4, 5), thus leading to malignant transformation and metastasis. Among MET-associated cancers, TPR-MET translocation was found to be involved in the development of stomach adenocarcinoma (STAD) (6). Go H et al. reported the overexpression and amplification of MET in lung cancer, and increased MET expression is significantly associated with poor prognosis (7, 8). Moreover, in esophageal carcinoma (ESCA) and kidney renal papillary cell carcinoma (KIRP), gene amplification with consequent protein overexpression and constitutive kinase activation of MET has been reported (9, 10).

Because of its important roles in tumors, MET is considered to be a critical target for anticancer therapy. Multiple MET inhibitors have already been elaborated and tested in preclinical and clinical studies (11, 12). Especially in lung cancer, inhibition of MET receptor activity has shown promising results and has become a standard therapy for patients (13, 14). However, the treatment effects of MET inhibitors on other cancer types are less certain.

As previous research about MET aberrations in cancer is limited to the limited sample size and/or to the individual cancer type, a comprehensive profiling across different cancer types to explore their significance has not been studied. In this study, we first profiled the expression, mutations, and copy number variants (CNVs) of MET across 32 cancer types from The Cancer Genome Atlas (TCGA) datasets. Then, survival analysis was conducted to further examine the aberration patterns and potential clinical significance of MET in distinct tumors. Taken together, these findings highlight the important roles of MET in tumorigenesis and present promising targetable pathways and clinical opportunities for cancer research.

## Materials and Methods

### Data Acquisition and Reanalysis Using Different Bioinformatics Tools

MET expression in normal tissues was extracted from The Genotype-Tissue Expression (GTEx,^1^), which collects transcriptome data in a wide variety of tissue types from healthy individuals (15). MET mRNA expression data in different cancers were obtained from cBioportal^2^, which is an open web resource for exploring, visualizing, and analyzing multidimensional cancer genomics and clinical data (16). Furthermore, the expression data was generated from normalized values with the reference population of all samples independent of sample diploid status, termed as NormalizeExpressionLevels _allsampleref.py. A total of 10,953 patients with 10,967 samples across 32 cancer types were analyzed (Supplementary Table S1), and the mRNA expression data were log10 transformed. Next, we compared MET mRNA expression between tumors and their paired normal tissues using Gene Expression Profiling Interactive Analysis (GEPIA,^3^), a web server for cancer and normal gene expression profiling and interactive analyses (17). Then, we further explored MET protein expression using the level 4 TCGA RPPA dataset downloaded from The Cancer Proteome Atlas (TCPA,^4^) (18).

The cBioportal is a portal that enables users to interactively investigate genetic alterations across samples, genes, and, when available in the underlying data, to link these to clinical outcomes (16). In this study, mutation data, CNV data, and clinical data were downloaded from cBioportal. The mutation data consist of indels and SNVs. For the CNV data, the log ratio value means: −2 = deep deletion; −1 = shallow deletion; 0 = diploid; 1 = gain; 2 = amplification.

The clinical data were used to perform the survival association analysis for MET alteration or for MET amplification status. In addition, we mainly evaluated several survival indexes, such as overall survival (OS) and progression-free survival (PFS) (19–22). The association between MET expression and patients’ OS and PFS was analyzed using the Kaplan–Meier Plotter^5^, an open source platform that can be used to assess the effect of genes on patient survival. Moreover, Kaplan–Meier plotter is established by a PostgreSQL server, which can simultaneously integrate the clinical data and gene expression from several databases, such as GEO, EGA, and TCGA (23). The hazard ratio and 95% confidence intervals were presented as forest plots.

### Statistical Analyses

The statistical analyses were performed with SPSS 12.0 software (IBM Analytics, United States). Student’s *t*-test, Cox regression analysis, and linear regression analysis were performed when appropriate. *P* < 0.05 was defined as statistically significant. In addition, the statistic calculations on the Mutual Exclusivity tab are conducted using all cancer samples in cBioPortal. A sample is defined as altered or unaltered (controls) for each gene based on the Onco Query Language (OQL) utilized in the query. Especially, in single- and cross-cancer queries, OQL algorithm can be utilized to accurately identify copy number alterations, mutations, mRNA, and protein expression profiles (16).

## Results

### MET Expression in Pancancer

**FIGURE 1:**
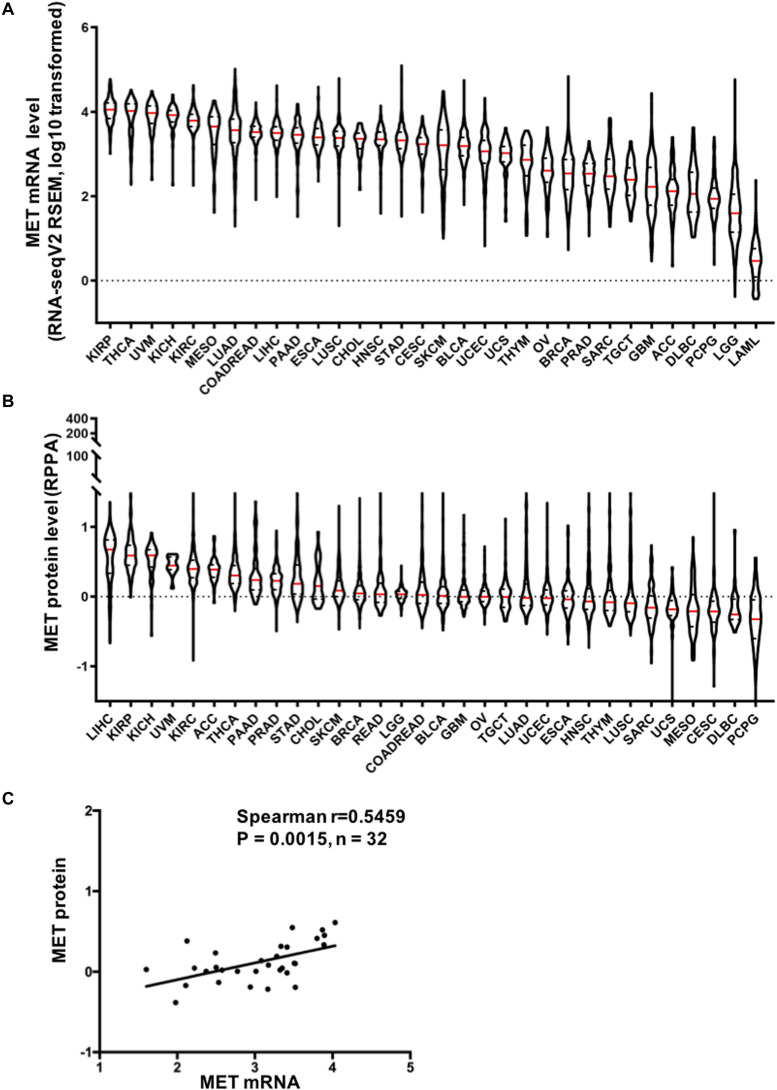
Mesenchymal–epithelial transition factor (MET) mRNA and protein expression in The Cancer Genome Atlas (TCGA) cancer tissues. **(A)** MET mRNA expression (RNA-seqV2 RSEM, log10 transformed) across 32 cancer types. **(B)** MET protein expression (RPPA, replicate-based normalized) in TCGA cancer tissues. Sample lines represent medians and quartiles. All TCGA abbreviations are shown in Supplementary Table S1. **(C)** MET mRNA expression was positively correlated with MET protein expression in TCGA cancer tissues. *n* = 32 indicates the 32 types of cancer in TCGA.

MET overexpression has been reported in many human cancers (24–26). Previous research on MET expression in cancer is limited to the small sample size and/or to the limited number of cancer types. Here, we provide a more comprehensive evaluation of MET expression in pancancer. First, we extracted data from the GTEx portal and analyzed MET expression in 53 types of normal tissues. As shown in Supplementary Figure S1A, MET expression among different tissues was dramatically different. The tibia had the highest expression. Whole blood and EBV-transformed lymphocytes had almost no MET expression. Then, we compared MET mRNA expression across 32 TCGA cancer types. MET expression showed quite a broad spectrum, suggesting that cancers with highly expressed MET may have unique genetic features that promote increased MET expression. Based on the interquartile range, some cancer types, such as lower-grade glioma (LGG), breast invasive carcinoma (BRCA), and glioblastoma multiforme (GBM) have a widespread of MET expression, while cholangiocarcinoma (CHOL) has a narrow spread, which may be due to some cancer types having more than one subtype and therefore having more genetic diversity (Figure 1A). Moreover, we compared MET mRNA expression between tumors and their paired normal tissues profiled in TCGA. Significantly differential expression was found in 23 cancer types, with three cancer types downregulated [BRCA, acute myeloid leukemia (LAML), and LGG] and 20 cancer types upregulated [cervical squamous cell carcinoma and endocervical adenocarcinoma (CESE), colon adenocarcinoma (COAD), lymphoid neoplasm diffuse large B-cell lymphoma (DLBC), ESCA, head and neck squamous cell carcinoma (HNSC), kidney chromophobe (KICH), KIRP, LUAD, lung squamous cell carcinoma (LUSC), ovarian serous cystadenocarcinoma (OV), pancreatic adenocarcinoma (PAAD), rectum adenocarcinoma esophageal carcinoma (READ), skin cutaneous melanoma (SKCM), STAD, testicular germ cell tumors (TGCT), thyroid carcinoma (THCA), thymoma (THYM), uterine corpus endometrial carcinoma (UCEC), and uterine carcinosarcoma (UCS)] (Supplementary Figure S1B). The cancer type with the most decreased expression was BRCA with 2.1 TPM (tumor) compared to 3.4 TPM (normal tissue). The cancer type with the most increased expression was ESCA with 5.0 TPM (tumor) compared to 1.5 TPM (normal tissue).

In addition, we also compared MET protein expression across 32 TCGA cancer types using data from TCPA. Similar to MET mRNA, MET protein expression exhibited quite a broad spectrum of expression levels and varied across different cancer types (Figure 1B). Moreover, MET mRNA and protein expression levels were highly correlated and positively associated in pancancer (*r* = 0.5459, *p* = 0.0015), indicating a critical role for gene expression regulation in driving the protein expression of MET and its functional status in the tumor (Figure 1C).

### MET Somatic Mutation Patterns Across Cancer Types

**FIGURE 2:**
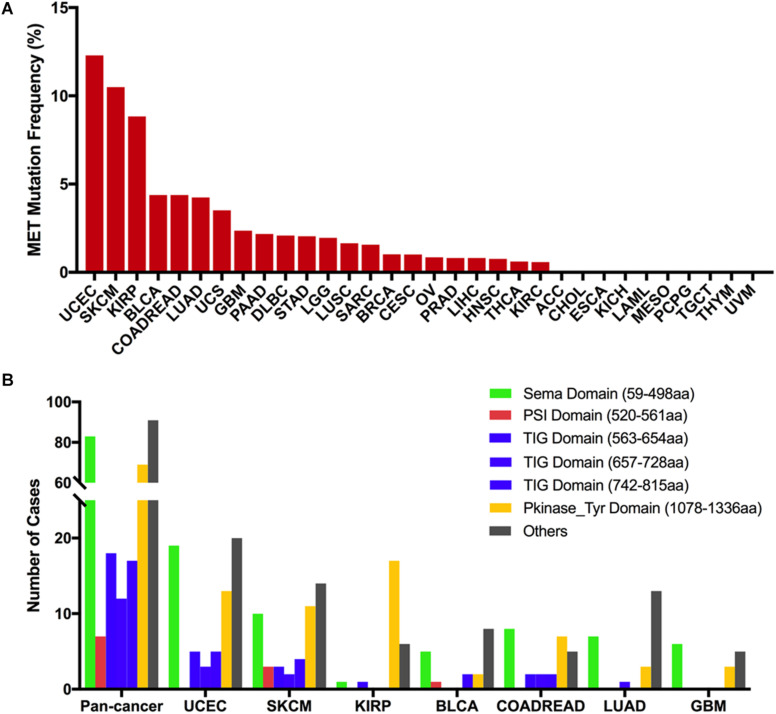
MET mutation distribution in TCGA cancer tissues and protein functional domains. **(A)** MET mutation frequency across 32 cancer types. **(B)** MET mutation distribution in different functional domains for all cancer types together and for the top seven cancer types. Abbreviations: aa, amino acid.

Across the 32 cancer types, the total mutation frequency of MET was 2.3% (251/10,953) for all patients and 2.8% (311/10,967) for all cancer samples. MET mutations were observed most commonly in UCEC (12.3%), SKCM (10.5%), KIRP (8.8%), bladder urothelial carcinoma (BLCA, 4.4%), COADREAD (4.4%), and LUAD (4.2%). In contrast, however, adrenocortical carcinoma (ACC), CHOL, ESCA, KICH, LAML, mesothelioma (MESO), pheochromocytoma and paraganglioma (PCPG), TGCT, THYM, and uveal melanoma (UVM) virtually barely had MET mutations (Figure 2A). The number of samples from each cancer type ranged from 36 (CHOL) to 1,084 (BRCA), and those having too few samples might not accurately reflect the full picture of MET mutation status (Supplementary Table S2).

Based on the Pfam database, MET harbors six functional domains, including the Sema (59–498 aa), PSI (520–561 aa), TIG (563–654 aa), TIG (657–728 aa), TIG (742–815 aa), and PKinase-Tyr domains (1,078–1,336 aa). The Sema domain, a seven-bladed β-propeller semaphorin domain, could lead to receptor oligomerization when binding to ligand (27); the PSI domain is a cysteine-rich region and represents short regions of secondary structure including two α-helices and a three-stranded antiparallel β-sheet (28); the TIG domains are a region that has an immunoglobulin (Ig)-like fold (29); the PKinase-Tyr domain executes the phosphorylation function and controls the kinase activity of MET (30, 31). Here, we found that there were 311 MET somatic mutations across 32 cancer types, and all these mutations were broadly distributed across different functional domains of the MET gene. The most common domains were the other domain (91 samples), Sema domain (83 samples), Pkinase-Tyr domain (69 samples), TIG domain (563–654 aa, 18 samples), TIG domain (742–815 aa, 17 samples), TIG domain (657–728 aa, 12 samples), and PSI domain (7 samples). Meanwhile, the location distribution of MET somatic mutations was quite different across all cancer types. Mutations in UCEC, SKCM, BLCA, and LUAD were most commonly located in the other domain whose functions were barely known. Mutations in KIRP were primarily located in the Pkinase-Tyr domain, approximately three times more than the mutations located in the other domain. Mutations in COADREAD and GBM were mainly located in the Sema domain (Figure 2B and Supplementary Table S3).

The 311 MET somatic mutations could be divided into four categories according to their functional impact on protein coding. Missense mutation was the most common type of mutation (250 mutations), followed by truncating mutation (46 mutations), other mutation (14 mutations), and in-frame mutation (one mutation) (Supplementary Figure S2A). The most frequent mutation positions were 1,010 aa in the other domain and 1,148 aa in the Pkinase-Tyr domain. For example, the 1,010-aa mutation was found in seven samples (six samples with X1010 splice, one with D1010fs) and occurred almost exclusively in LUAD (6/7) (Supplementary Figure S2B). MET X1010_splice alteration is known to be oncogenic, and LUAD patients harboring the MET X1010 splice can be treated with the NCCN-compendium listed drug crizotinib (32, 33) and FDA approved capmatinib (34, 35). Meanwhile, tepotinib, a MET inhibitor, was also approved in Japan in March 2020 for the treatment of LUAD patients harboring MET exon 14 skipping (36, 37). The only other tumor with mutations at this position was LGG (one sample with X1010_splice), but its role was almost unknown to this cancer. The 1,148-aa mutation in the Pkinase-Tyr domain was also observed in seven samples [six samples with R1148Q (three SKCMs, one BLCA, one BRCA, one COADREAD), one sample with R1148^∗^ (one UCEC)]. However, the oncogenic function of mutations at this position was considered unknown, and there were no FDA-approved treatments specifically for patients with these mutations. The most mutated positions in KIRP (17 of 25 mutations) were located at the Pkinase Tyr domain, especially at the 1,250-aa position (four samples with M1250T) and the 1,092- to 1,094-aa position (three with V1092I, three with H1094Y). The mutation at these positions was known to be oncogenic (Supplementary Figure S2C). UCEC harbored the highest frequency of MET mutational alterations; however, the oncogenic function of these mutations was largely unknown. The most mutated positions in UCEC (3 of 78 mutations) were located at the Pkinase-Tyr domain at the 1,186-aa position (one with L1186F, one with L1186I, one with L1186R), but its oncogenic role was considered unknown. D1228Y/A and T222K alterations were found in UCEC (one with D1228Y, one with D1228A, one with T222K) and known to be likely oncogenic and predicted oncogenic, respectively (Supplementary Figure S2D).

**FIGURE 3:**
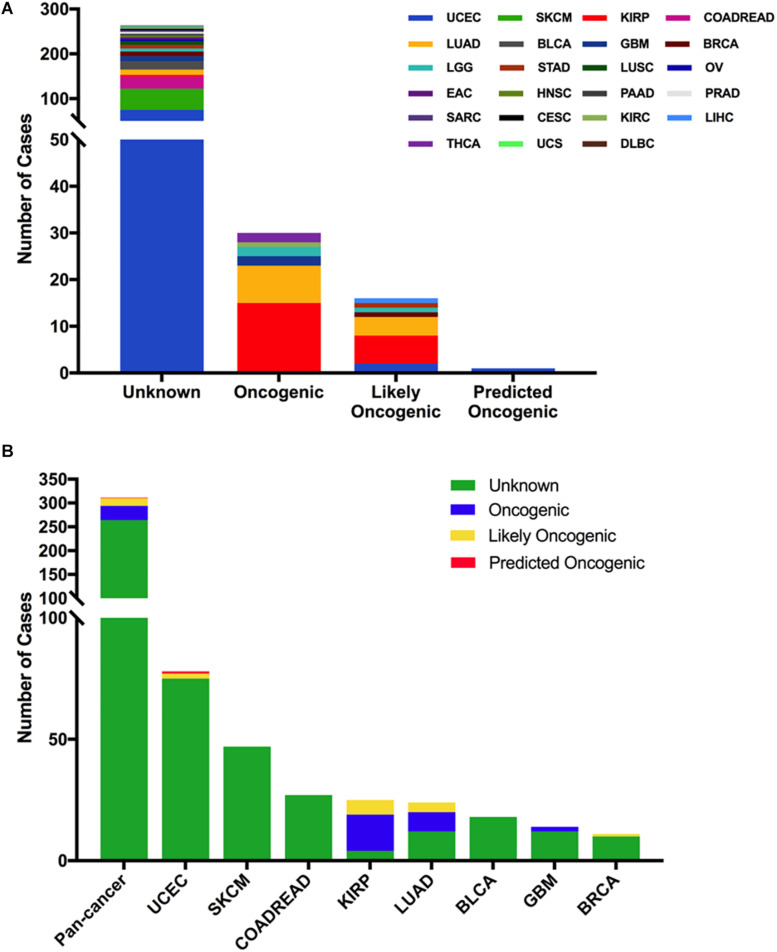
MET mutation classification according to the functional impact on protein coding. **(A)** MET mutation classification according to the functional impact on all tumors together. **(B)** Functional impact class distribution of MET mutations in all and top eight cancer types.

From oncogenic effect and predictive significance, these 311 somatic mutations were classified into four categories: oncogenic (30 mutations), likely oncogenic (16 mutations), predicted oncogenic (one mutation), and unknown (264 mutations). Most of these mutations belonged to the unknown class, suggesting that more efforts are needed to determine the meanings of these mutations (Figure 3A). However, in some types of cancer, like KIRP and LUAD, the MET mutations were mainly distributed in the functional categories. As shown in Figure 3B, 60% (15/25) of the mutations in KIRP were oncogenic, followed by likely oncogenic (6/25) and unknown (4/25). In LUAD, half of the mutations were oncogenic/likely oncogenic (12/24).

**FIGURE 4:**
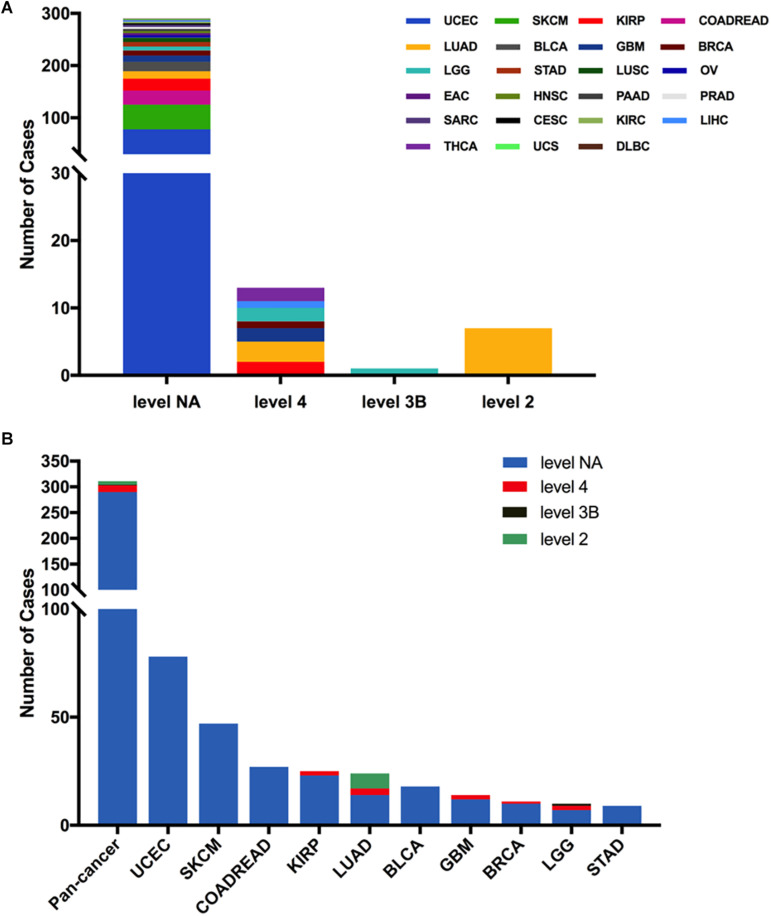
MET mutation distribution according to targeted therapy implications. **(A)** MET mutation distribution according to the clinical targeted therapy implications as annotated in OncoKB among all cancer types together. **(B)** Targeted therapy implication distribution of MET mutations in all and top 10 cancer types.

Next, we analyzed the clinical targeted therapy implications of MET mutation using cBioPortal, which could provide the annotation of variants from different databases, including COSMIC, Cancer Hotspots method, CIViC, My Cancer Genome, and OncoKB. However, the exact levels of clinical actionability displayed in cBioPortal can be fully defined using OncoKB (16). Thus, for the clinical targeted therapy implications, each MET somatic mutation could be classified into four levels as defined by OncoKB (38): level 2 (seven mutations), level 3B (one mutation), level 4 (13 mutations), and level NA (290 mutations) (Figure 4A and Supplementary Table S2). Only level 2 was represented for targeted therapy with an NCCN-compendium listed drug (39). All level 2 mutations were observed in LUAD (6 × 1010_splice, 1 × 1009_splice). In LUAD, half of the mutations were oncogenic/likely oncogenic, and over half of them were in level 2 (7 of 12 mutations). However, KIRP had the highest proportion of oncogenic/likely oncogenic mutations (21 of 25 mutations), and almost all were in level NA without targeted therapy implications (Figure 4B).

### MET CNVs in Different Cancer Types

**FIGURE 5:**
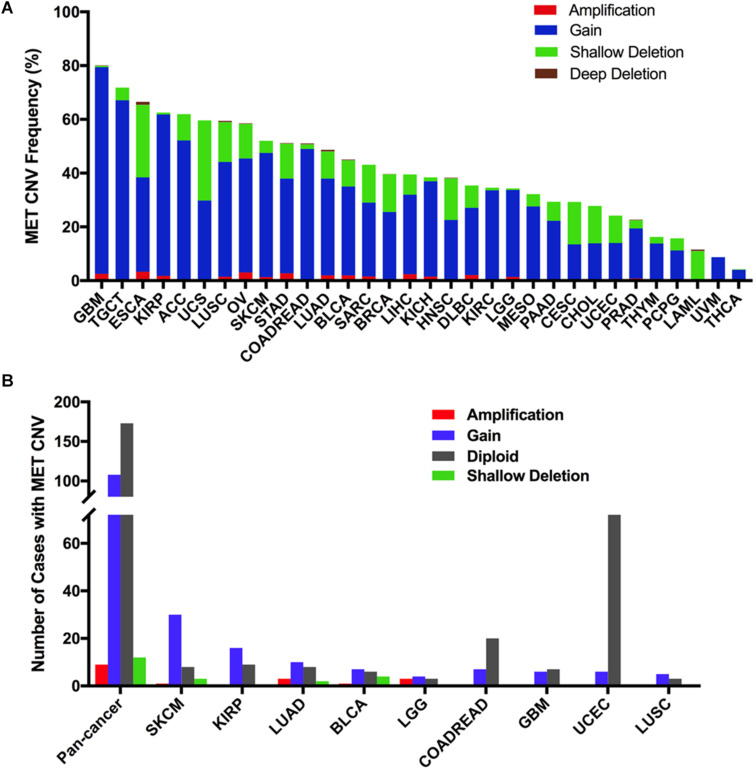
MET copy number variant (CNV) distribution in TCGA cancer tissues. **(A)** MET CNV frequency across 32 TCGA cancer types. **(B)** MET CNV distribution for all cancer types together and for the top nine cancer types. Abbreviations: CNV, copy number variant.

Across the 32 cancer types, the total MET CNV frequency was 42.1% (detected in 4,622 of 10,967 samples). Most of them were gained (3,577 samples), followed by amplification (129 samples), shallow deletion (899 samples), and deep deletion (17 samples). The most common tumors with MET CNVs were GBM (80.1%), TGCT (71.8%), ESCA (66.5%), KIRP (62.5%), and ACC (62.0%). In contrast, PCPG (15.7%), LAML (11.5%), UVM (8.8%), and THCA (4.2%) showed very low MET CNV frequencies (Figure 5A). To determine whether MET CNVs were associated with MET expression, we compared MET CNVs with MET mRNA expression across 32 TCGA cancer types. The results showed that MET CNVs and mRNA expression were highly correlated in pancancer (*r* = 0.1511, *p* < 0.0001) (Supplementary Figure S3), indicating important roles for gene copy number in determining MET mRNA expression and its functional status in the tumor. Among the 311 samples with MET mutations mentioned above, 129 also harbored MET CNVs (108 with gain, nine with amplification, and 12 with shallow deletion). SKCM and KIRP had the highest gain among different cancer types (Figure 5B and Supplementary Table S2). As shown in Figure 5A and Figures 1A,B, KIRP harbored a very high proportion of gain and was also the cancer type with higher MET expression. Likewise, PCPG, which lacked the amplification and gain of MET, had a lower level of MET mRNA and protein expression. However, some cancer types lacked the amplification and gain of MET but had a high level of MET expression (e.g., UVM and THCA), suggesting that additional genetic alterations could contribute to high expression of MET in the tumor.

### Combined MET Alterations (Mutation and CNVs) in Different Cancer Types

**FIGURE 6:**
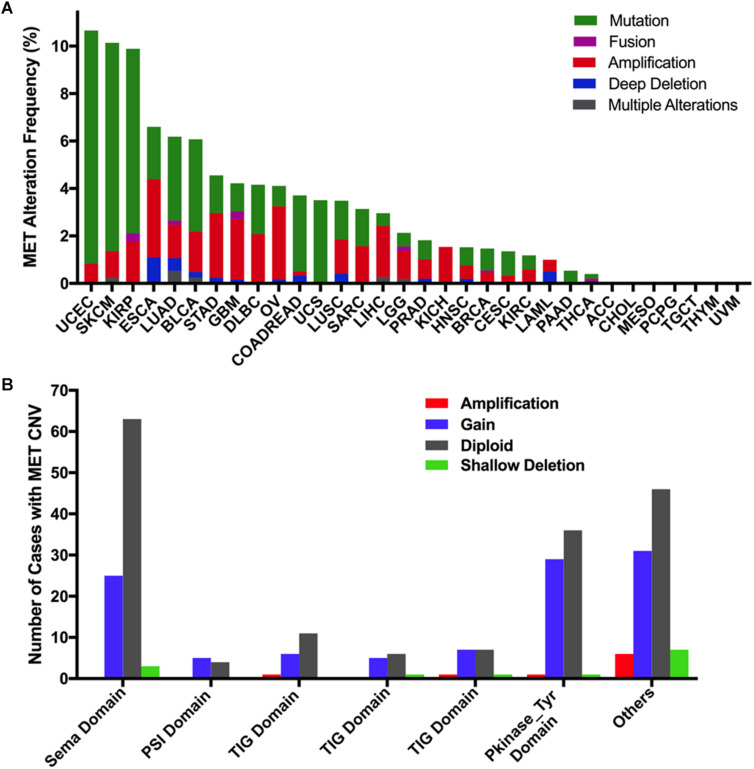
MET alteration distribution in TCGA cancer tissues. **(A)** MET alteration (combined mutation and CNVs) frequency across 32 cancer types. **(B)** The distribution of MET CNVs along with mutations located in different protein functional domains of MET.

Overall, the combined MET mutation and CNV frequency in all cancer types were observed in 3.6% of cases (390 of 10,967 samples). However, MET alterations are quite variable across different cancers. MET alterations were observed most commonly in UCEC (10.21%), SKCM (10.14%), and KIRP (9.89%), in which mutations were more common. Other cancer types with dominant MET mutations but at much lower mutation rates included LUAD (3.53%), BLCA (3.89%), COADREAD (3.2%), UCS (3.51%), and PAAD (0.54%). Furthermore, tumors such as ESCA, STAD, GBM, and OV mainly had MET CNVs but relatively few mutations (3.3 vs 2.2%, 2.73 vs 1.59%, 2.53 vs 1.18%, 3.08 vs 0.86%, respectively). Tumors including ACC, TGCT, THYM, UVM, MESO, CHOL, and PCPG had neither MET CNVs nor MET mutations (Figure 6A).

Interestingly, CNVs and mutation location were found to be associated. Approximately half of the mutations (29 of 67 mutations) in the Pkinase-Tyr domain also had MET copy gain, while nearly half of the mutations (44 of 90 mutations) in the other function-unknown domain were accompanied by amplification, gain, and shallow deletion. Mutations in the PSI domain and the TIG domain had very few CNVs (Figure 6B).

### MET Alterations and Patient Survival

**FIGURE 7:**
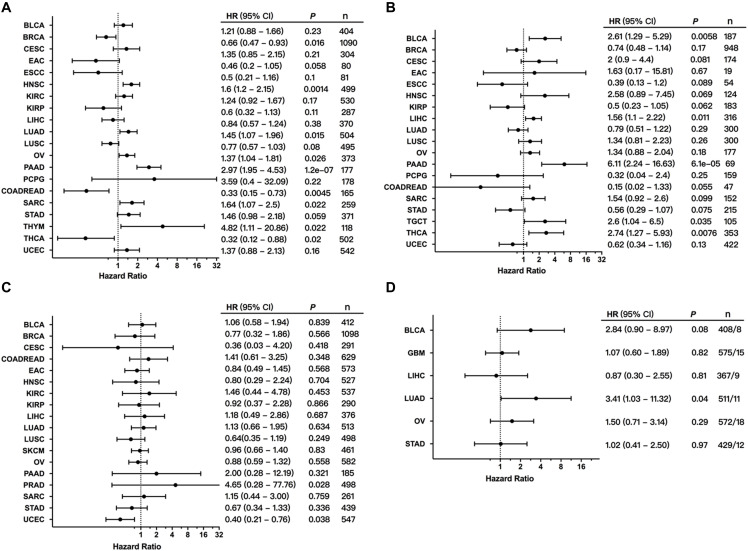
Association between MET alterations and patient prognosis. **(A)** The association between MET expression and patient overall survival (OS) as presented in the forest plot. **(B)** The association between MET expression and patient progression-free survival (PFS) as presented in the forest plot. **(C)** The association between MET alterations and patient OS as presented in the forest plot. **(D)** The association between MET amplification and patient OS as presented in the forest plot. Only cancer types with at least eight tumor samples containing amplification were analyzed.

To explore the clinical significance of MET expression, we analyzed the association between MET mRNA expression and patient OS and PFS in individual cancer types. The results showed that high MET expression was associated with poor patient OS in HNSC, LUAD, OV, PAAD, sarcoma (SARC), and THYM. However, among patients with BRCA, READ, and THCA, high MET expression was associated with better patient OS (Figure 7A). Meanwhile, the association analysis between MET expression and patient RFS in individual cancer types showed that high MET expression was associated with short patient RFS in BLCA, LIHC, PAAD, TGCT, and THCA (Figure 7B).

We further explored the clinical significance of the MET alteration, and survival association analysis regarding alteration status in each cancer type showed that MET alteration was associated with short survival in PRAD. However, MET alteration was found to be associated with a better prognosis in UCEC (Figure 7C). This opposite result could be due to different genetic backgrounds and insufficient sample sizes. Moreover, when the survival association analysis was performed only for MET mutation status, MET mutations were associated with poor prognosis in LUAD (Figure 7D).

## Discussion

Our study profiled the characteristics of MET in 32 cancer types and showed that MET expression, mutation, and CNVs varied across different cancer types, which are of great clinical significance. UCEC, SKCM, and KIRP had the highest MET alteration, and mutations accounted for the major proportion. While mutations in UCEC and SKCM were most commonly located in the Sema domain and the other function-unknown domain, mutations in KIRP were primarily located in the Pkinase-Tyr domain, which is more important for treatment selection. Furthermore, although studies have shown a high MET expression in UCEC, SKCM, and KIRP, no association was observed between MET expression and patient prognosis. Other cancer types, including LUAD, BLCA, COADREAD, and UCS harbored similar characteristics; all their alteration frequency was between 4 and 6%, and mutation was the primary alteration. Mutations in LUAD are mainly X1010_splices, which are in exon 14, and mutations in this region are known for targeted therapy in clinical practice in NSCLC (33). On the other hand, tumors including KICH, OV, and LIHC mainly had MET CNVs but rarely mutations. Moreover, ACC, TGCT, THYM, UVM, MESO, CHOL, and PCPG rarely had MET alterations.

In recent years, splice site mutations have been discovered in MET, leading to exon 14 skipping. NSCLC patients who were carrying this splice variant typically overexpressed MET and showed a response to MET small molecule inhibitors such as crizotinib and cabozantinib (40). In this study, X1010_splice was found to be the most frequent mutation type in LUAD. This splice variant was located in the intracellular juxtamembrane domain. The juxtamembrane domain is encoded in part by MET exon 14 and contains several important regulatory elements, including the c-Cbl binding site, which contributed to the degradation of MET protein (41). MET X1010 splice alteration is known to be oncogenic, and LUAD patients harboring MET exon 14 alterations, such as MET X1010_splice, can be treated with the NCCN-compendium listed drug crizotinib (32, 33). However, several recent reports have shown that many patients receiving these MET small molecule inhibitors showed progression, and further studies to understand the resistance mechanisms are required (42). In addition, the prognostic role of MET in LUAD was quite clear, and both high expression and amplification of MET were significantly associated with poor prognosis. Moreover, in LUAD, half of the mutations were oncogenic/likely oncogenic, but half of them are still unknown, highlighting the challenge of further interpretation of mutations.

The MET pathway was reported to play an important role in KIRP (43). In this large TCGA dataset, KIRP had very high MET-combined alterations, and this high alteration was mostly driven by a high proportion of mutations. Compared with other cancer types, mutations in KIRP were primarily located in the Pkinase-Tyr domain, which is known for targeted therapy with TKIs. Currently, several MET TKIs, such as crizotinib, have been approved in NSCLC, but their applications in KIRP are still under active investigation (44). Consistent with the results found in our study, almost all mutations in KIRP were in level NA without targeted therapy. Patients’ outcomes were typically worse in KIRP when treated with conventional therapies, driving an urgent need for continued investigation on MET target therapy (43, 45). In addition, high expression of MET was discovered in KIRP, and most mutations in KIRP were oncogenic and likely oncogenic; however, there was no association observed between MET expression and patient prognosis in this dataset, although some reports indicated otherwise (46). This paradox could be due to the absence of well-known responsive mutations and the presence of alternative compensatory pathways interacting with MET pathways, such as the MAPK/ERK and PI3K/AKT pathways (43, 47, 48), which inspired further research on combinatorial therapy strategies in KIRP.

The patients with UCEC had the highest frequency of MET alterations, which was mostly driven by a high proportion of mutations. However, the detailed functional roles of these mutations were unknown. It is well known that genomic instability and high mutation rates cause cancer to acquire numerous mutations during evolution. Most are termed passenger mutations, which represent approximately 97% of all cancerous mutations and do not confer cancer phenotypes. Driver mutations are usually defined as mutations that give cancer cells a fundamental growth advantage for its neoplastic transformation (49). However, several recent reports have showed that passenger mutations may also have critical functional roles in driving cancer, with some authors describing them as mini drivers. They found that the aggregated impact of putative passenger mutations could provide significant predictive power to distinguish cancer from non-cancer phenotypes (50, 51). The above content implied to us that in some types of cancers, such as UCEC, even most of these mutations belonged to the unknown class; more efforts are needed to determine the meanings of these mutations, which might be found to also have important functional roles in driving tumorigenesis.

In addition, several reports have showed that some gene mutations, like BRAF mutation and ERBB2 mutation, were associated with MSI status in several cancer types (52–54). What is more, MET overexpression was also found to be associated with MSI status in gastric carcinomas (55). However, the association between MET alterations and MSI status in UCEC has not been reported yet and needs to be further clarified with more UCEC patients’ data. As we all know, the most frequent driver oncogenic mutations in SKCM were BRAF, NRAS, and KIT mutations (56), while the frequent MET mutation was rarely reported. However, high expression of MET was often reported in SKCM patients (57); the underlying mechanisms driving MET overexpression in SKCM is unknown. Especially, much more efforts are needed to further explore the roles of MET alterations in SKCM patients.

The prognostic roles of MET in PAAD were quite clear in this dataset. High MET expression was significantly associated with both short OS and PFS of patients with PAAD. However, PAAD harbored very few alterations in which mutations accounted for the most, and the meaning of these mutations is rarely known. Thus, more efforts to interpret these unknown mutations are needed.

Esophageal carcinoma, STAD, and OV harbored similar characteristics in this dataset: high expression of MET, high frequency of MET CNV, but relatively few MET mutations. Multiple therapeutic agents that target the hepatocyte growth factor (HGF)–MET pathway in these cancers are under development (58). Targeted therapy with rilotumumab, an anti-HGF IgG2 antibody that inhibits HGF–MET binding (59), along with traditional therapy could extend OS and PFS in MET-positive patients with ESCA and STAD in a phase II study (60) but failed in phase III clinical trials (58). Moreover, for patients with OV, phase II trials using rilotumumab had been conducted and showed limited impact on patient survival (61, 62). Thus, additional research on targeted therapy in these cancers is required.

Nevertheless, there were some limitations that need to be noted in this study. First, although we profiled 32 cancer types, some cancer types did not have sufficient sample size, leading to the full expression and alteration spectrum of MET being hard to achieve. More studies with sufficient samples in these cancers should be investigated further. Additionally, the alteration frequency of MET across all cancer types was approximately 0 to 10%. This low alteration frequency also made our analysis more difficult and challenging. Moreover, we mainly focus on the pancancer analysis of MET expression and alterations across multiple cancer types, without in-depth analysis for individual cancer types. In addition, though several papers have reported the alteration profiles of MET in human cancers (12, 63, 64), these results among the published data might be biased due to additional curation during the publication process. Thus, in our reports, the MET profiles were mainly evaluated by cBioportal, which could unify the TCGA data across all tumor types with uniform clinical elements and ideally processed curation (65).

## Conclusion

In conclusion, we first reported the comprehensive pancancer views of MET aberrations and their association with patient outcomes across 32 TCGA cancer types. Some alterations are more involved in the development of tumors, while others participate more in targeted therapy. Moreover, some cancer types with low MET alteration frequency were associated with outcomes, but unexpectedly, others with high alteration frequency were not. Taken together, these results provide a significant novel understanding of MET deregulation in cancer biology. The data presented here are also relevant for targeting MET in cancer therapy, both by revealing vulnerable cancer types and by identifying potential therapeutic biomarkers.

## Data Availability Statement

All datasets presented in this study are included in the article/Supplementary Material.

## Author Contributions

YY and ZX contributed to the conception and design of this study. JL, KH, YY, and ZX contributed to writing, review, and/or revision of the manuscript. LZ, JH, and SZ provided administrative, technical, and/or material support. All authors approved the final version of manuscript.

## Conflict of Interest

The authors declare that the research was conducted in the absence of any commercial or financial relationships that could be construed as a potential conflict of interest.

## Footnotes

1 https://www.gtexportal.org/

2 http://cbioportal.org/

3 http://gepia.cancer-pku.cn/

4 http://tcpaportal.org

5 http://kmplot.com/

## Supplementary Material

Supplementary Figure 1 MET expression in normal and cancer tissues. **(A)** MET expression among 53 types of normal tissues. **(B)** MET expression between tumors and paired normal samples across TCGA cancer types from GEPIA. MET expression is upregulated in CESE, COAD, DLBL, ESCA, HNSC, KICH, KIRC, KIRP, LUAD, LUSC, OV, PAAD, READ, SKCM, STAD, TGCT, THCA, THYM, UCEC, and UCS but downregulated in BRCA, LAML, and LGG (all *P* < 0.01). Abbreviations: TPM, transcripts per million.

Supplementary Figure 2 MET mutation distribution in different protein functional domains. **(A)** MET mutation distribution in different protein functional domains for all cancer types together. **(B)** MET mutation distribution in different protein functional domains in LUAD. **(C)** MET mutation distribution in different protein functional domains in KIRP. **(D)** MET mutation distribution in different protein functional domains in UCEC.

Supplementary Figure 3 The correlation between MET CNVs and MET mRNA expression. MET linear copy number value correlations with MET mRNA expression (RNA-seqV2 RSEM, log10 transformed) across different cancer types.

Supplementary Table 1 Summary of TCGA cancer types and sample size.

Supplementary Table 2 Detailed information on 311 human MET somatic mutations from cBioportal for 32 cancer types.

Supplementary Table 3 Somatic mutation distribution in different functional domains of MET for cancers with MET mutations and all cancers together.
