# Supplementary figures and images for: Spectrum of Mesenchymal–Epithelial Transition Aberrations and Potential Clinical Implications: Insights From Integrative Pancancer Analysis
Source: Front Oncol. 2020 Oct 15;10:560615. doi: 10.3389/fonc.2020.560615 (PMC7593712; doi:10.3389/fonc.2020.560615)

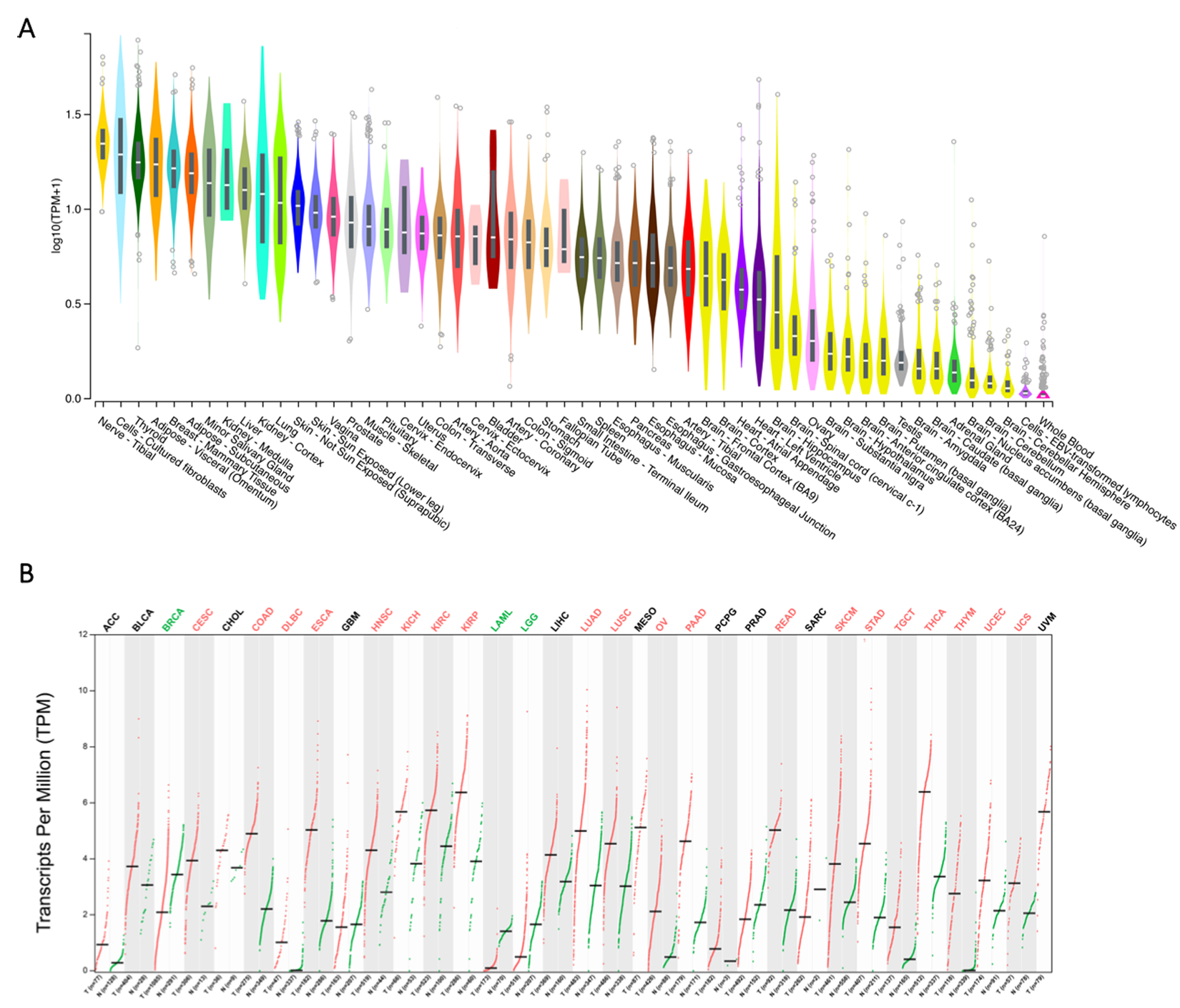

Supplement: Supplementary Figure 1 — MET expression in normal and cancer tissues. (A) MET expression among 53 types of normal tissues. (B) MET expression between tumors and paired normal samples across TCGA cancer types from GEPIA. MET expression is upregulated in CESE, COAD, DLBL, ESCA, HNSC, KICH, KIRC, KIRP, LUAD, LUSC, OV, PAAD, READ, SKCM, STAD, TGCT, THCA, THYM, UCEC, and UCS but downregulated in BRCA, LAML, and LGG (all P < 0.01). Abbreviations: TPM, transcripts per million. [file Image_1.TIF]

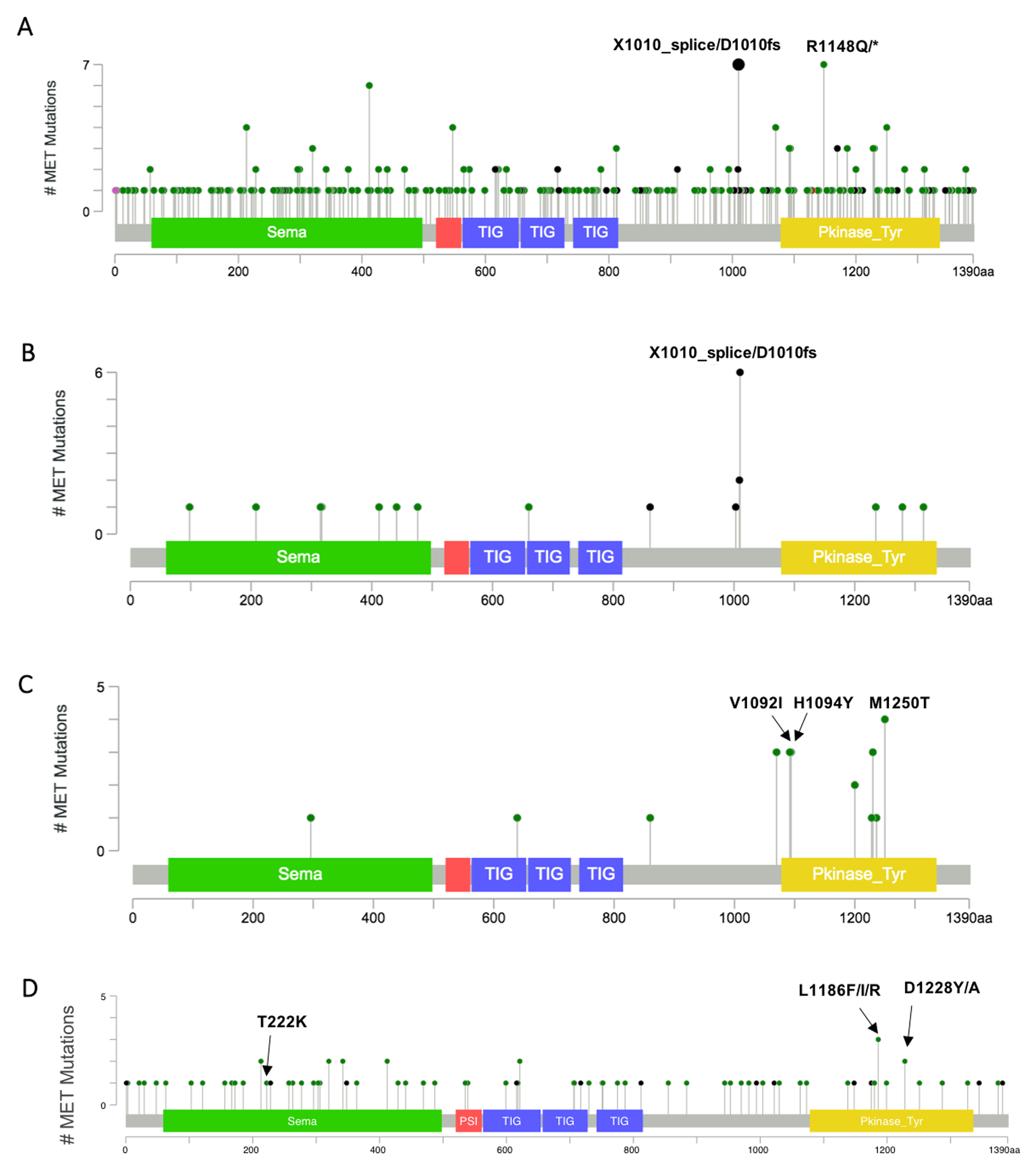

Supplement: Supplementary Figure 2 — MET mutation distribution in different protein functional domains. (A) MET mutation distribution in different protein functional domains for all cancer types together. (B) MET mutation distribution in different protein functional domains in LUAD. (C) MET mutation distribution in different protein functional domains in KIRP. (D) MET mutation distribution in different protein functional domains in UCEC. [file Image_2.TIF]

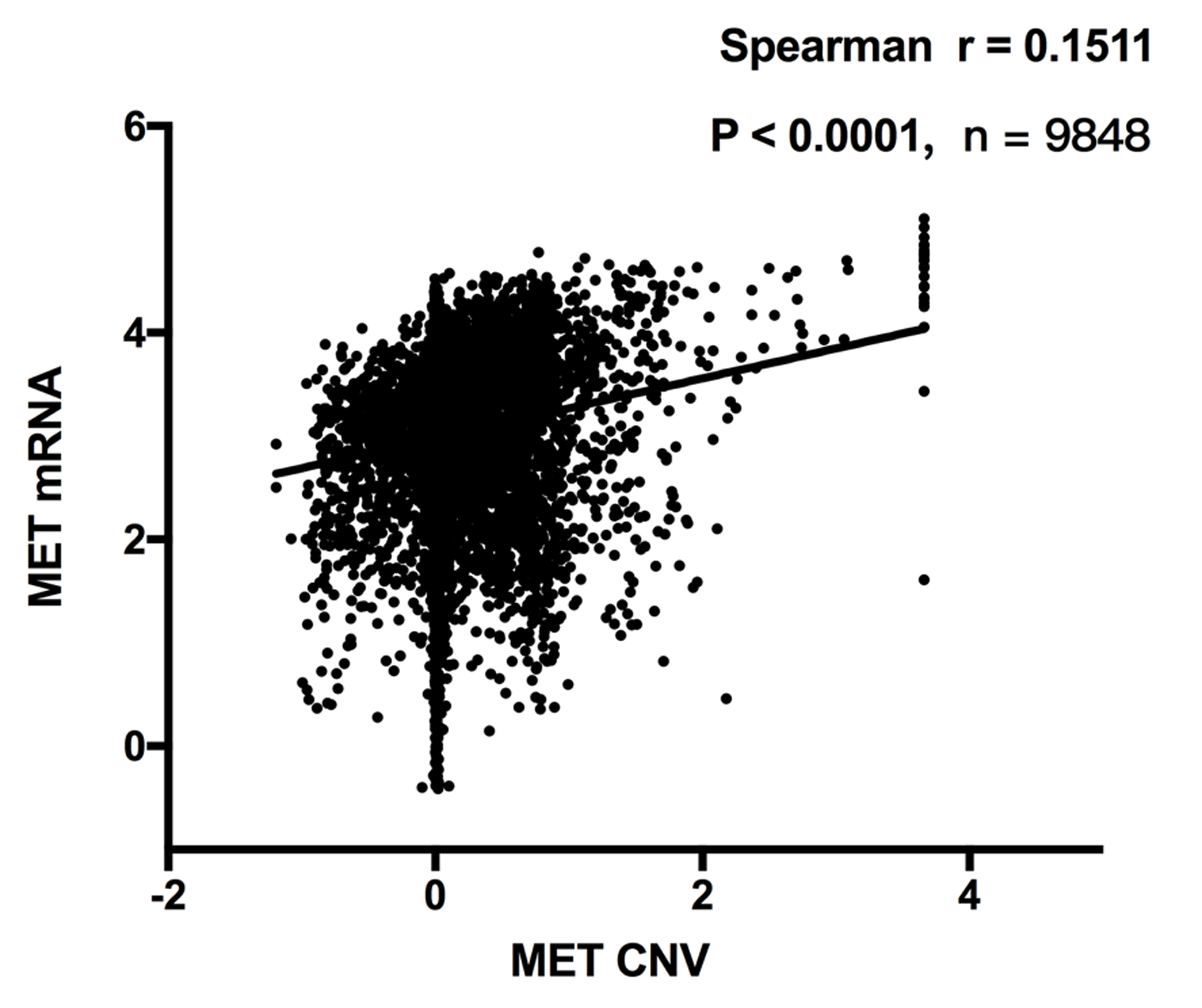

Supplement: Supplementary Figure 3 — The correlation between MET CNVs and MET mRNA expression. MET linear copy number value correlations with MET mRNA expression (RNA-seqV2 RSEM, log10 transformed) across different cancer types. [file Image_3.TIF]
